# Treatment Response and Prognosis of Primary Angiitis of the Central Nervous System: A Real‐World Observation

**DOI:** 10.1002/cns.70884

**Published:** 2026-04-17

**Authors:** Xin Liu, Ti Wu, Linlin Yin, Fu‐Dong Shi, Huabing Wang

**Affiliations:** ^1^ Department of Neurology, Beijing Tiantan Hospital Capital Medical University Beijing China; ^2^ Department of Neurology Tianjin Medical University General Hospital Tianjin China

**Keywords:** primary angiitis of the central nervous system, prognosis, real‐world study, therapy

## Abstract

**Subject:**

To analyze the treatment response in patients with primary angiitis of the central nervous system (PACNS) in a large vasculitis cohort.

**Method:**

In this single‐center retrospective observational study, we assessed treatment, relapses, remission, and outcome of patients with PACNS. We pooled the patients' relapses under different treatments as well as various immunotherapies. Multivariate logistic regression analysis was performed to determine factors independently associated with relapse and those associated with good functional status. The time of observation was 96 months.

**Result:**

The cohort comprised 80 patients, with 38 diagnosed with pathologically confirmed PACNS and 42 with clinically diagnosed PACNS, with a median follow‐up duration of 18 months (range 3–96). Treatment comprised acute‐phase induction therapy with high‐dose corticosteroids, alone or combined with immunosuppressive agents, followed by remission‐phase maintenance immunosuppressive therapy, primarily with cyclophosphamide, rituximab, or mycophenolate mofetil. Following treatment, 49 patients (61.3%) achieved remission and 70 (87.5%) attained favorable functional outcomes. The overall relapse rate was 35%. Group 3 demonstrated significantly higher baseline disease severity (*p* < 0.05). Multivariate analysis identified seizures and cognitive impairment as predictors of relapse.

**Conclusion:**

This study demonstrates that a majority of PACNS patients exhibit a favorable response to therapy. For patients presenting with more severe disease at diagnosis, long‐term maintenance therapy following remission induction with glucocorticoids or immunosuppressive agents is required.

## Graphical abstract

This study of a large Chinese cohort of PACNS clarifies real‐world treatment strategies, relapse patterns, and functional outcomes. The findings demonstrate most patients respond favorably to therapy. The overall relapse rate was 35%, with seizures and cognitive impairment identified as significant predictors of relapse.

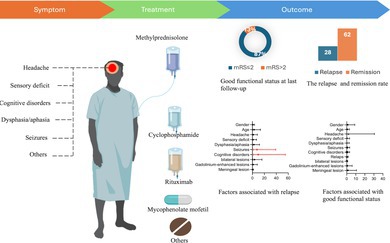

## 1 Introduction

Primary angiitis of the central nervous system (PACNS) is an inflammatory disorder of the central nervous system (CNS) characterized by an unexplained vasculitis confined to the brain, spinal cord, and leptomeninges [1]. In the absence of treatment, the disease course can result in morbidity and mortality. A small number of adult cohorts exist with different therapeutic management. Earliest reports suggested a poor outlook with fatal outcome in most patients, highlighting the critical need for effective treatment strategies [2, 3]. Previous studies suggest that aggressive therapeutic regimens can yield favorable prognostic outcomes for patients [4]. Conventional management typically involves corticosteroid therapy during acute relapses, with varying durations of maintenance therapy thereafter. Recent trends indicate that approximately 80% of patients in contemporary cohorts have been administered immunosuppressive agents, including cyclophosphamide (CYC), rituximab, and mycophenolate mofetil (MMF) [2, 5, 6]. However, significant gaps persist in understanding the optimal duration of these immunosuppressive therapies, therapeutic responses, and the long‐term prognostic implications associated with their use in China.

This research endeavors to conduct a comprehensive analysis of overall clinical data from patients with a diagnosis of PACNS at Beijing Tiantan Hospital. Our principal objectives are to assess treatment responses and all underlying favorable prognostic clinical factors, and to explore potential risk factors linked to relapses. Our research sheds insights into long‐term standard treatment regimen in enhancing the outcomes in PACNS.

## 2 Method

We conducted a retrospective study of patients diagnosed with PACNS according to the Calabrese and Mallek criteria at Beijing Tiantan Hospital from 2016 to 2024, which included 38 patients with pathologically confirmed PACNS and 42 patients with clinically probable PACNS [7]. Pathological diagnoses were defined according to the criteria of Athear Alrawi and Musch [8]. Long‐term follow‐up was performed on all included patients to evaluate disease progression, treatment outcomes, and clinical outcomes.

Patients were stratified into four treatment groups: Group 1 (*n* = 21), glucocorticoid monotherapy without immunosuppressant induction or maintenance; Group 2 (*n* = 6), induction therapy with glucocorticoids and immunosuppressants, but no maintenance; Group 3 (*n* = 41), combined glucocorticoid/immunosuppressant induction followed by maintenance therapy; Group 4 (*n* = 5), immunosuppressant‐based induction and maintenance without glucocorticoids.

### 2.1 Definition

Initial therapy was defined as the first treatment administered following an acute episode, consisting of either glucocorticoid monotherapy or glucocorticoid therapy in combination with immunosuppressive induction therapy. Maintenance therapy was defined as the immunomodulatory drug regimen selected by the patient for long‐term management following the initial treatment. Relapse was defined as a recurrence or worsening of symptoms, or progression of existing or new lesions on subsequent MRI examinations while the patient received no medication or a stable dose. A diagnosis of relapse required an inclined therapy. Remission was defined as the absence of manifestations of active PACNS after discontinuation of therapy for a minimum of 6 months. A modified Rankin Scale (mRS) score of less than 3 was defined as a Good Functional Status. Severe disease was defined as the condition in patients presenting with either a high risk of relapse or a mRS score more than 2.

### 2.2 Statistical Analysis

Data collection included demographic data, information about the clinical manifestation, initial cerebrospinal fluid findings, neuroimaging studies, histopathology of brain biopsy specimen, treatment regimen data (compounds, start/stop dates and dosages), onset and frequency of relapses with clinical and radiological information, and regular functional outcome measures. Categorical variables are expressed as numbers (%), and quantitative variables are expressed as medians (range). Categorical variables were analyzed with the *χ* ^2^ or Fisher exact tests as appropriate, and quantitative variables were analyzed with the Wilcoxon rank‐sum test. Univariate and multivariate logistic regression analysis was performed to determine factors independently associated with relapse and those associated with good functional status using variables. All *p* values were two‐sided; significance was defined at *p* < 0.05. The statistical analysis was performed using SPSS 24.

## 3 Result

### 3.1 Patients' Characteristics

**FIGURE 1:**
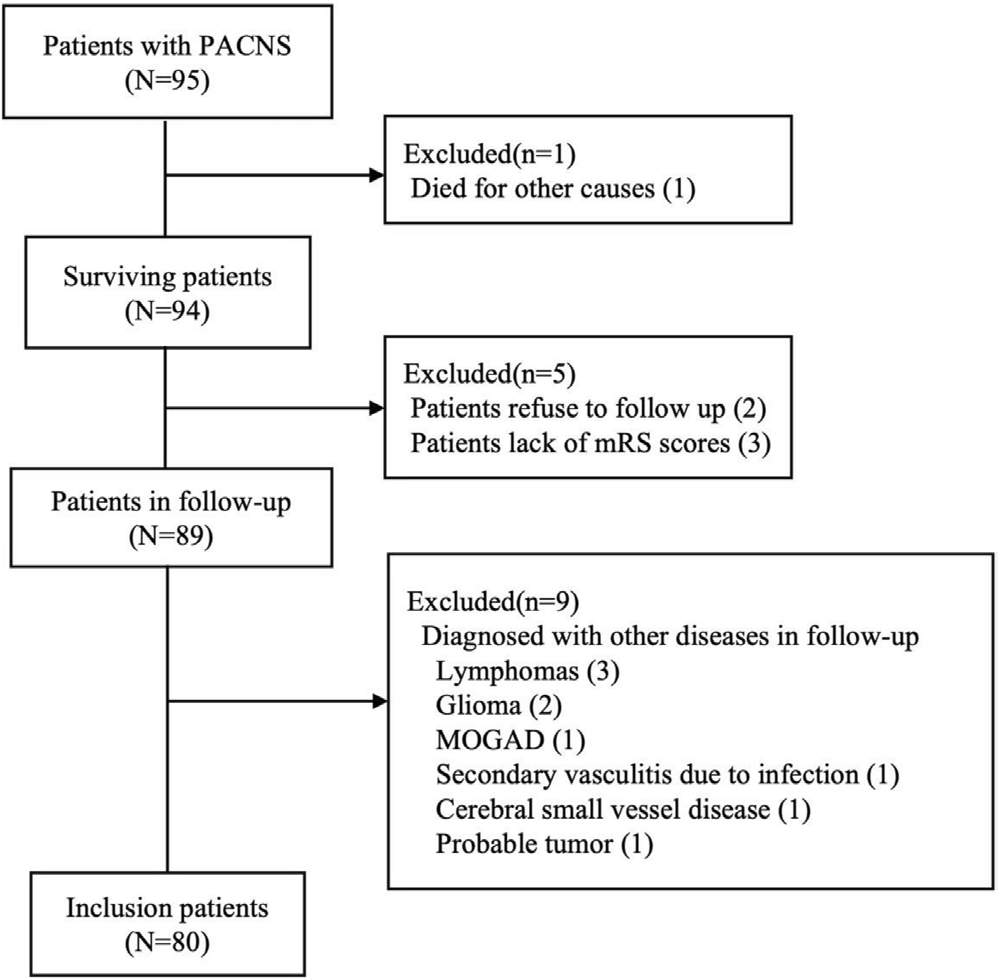
Illustrates the patient selection process and study enrollment flowchart.

The study cohort comprised 80 consecutively enrolled patients with confirmed diagnoses (Figure 1). Baseline demographic and clinical characteristics at initial presentation are summarized in Table 1. Neurological manifestations predominated, with dysphasia/aphasia constituting the most prevalent presentation (*n* = 47, 58.8%), followed by epileptic seizures (*n* = 31, 38.8%) and cognitive dysfunction (*n* = 22, 27.5%). Visual disturbances including reduced visual acuity and/or visual field deficits, were observed in 12 cases (15.0%). Histopathological confirmation was obtained in 38 cases (47.5%), with subtype distribution as follows: granulomatous vasculitis (*n* = 8, 21.1%), lymphocytic infiltration (*n* = 29, 76.3%), and necrotising vasculitis (*n* = 1,2.6%). Comprehensive vascular evaluation was completed in 58 participants (72.5%), employing multimodal imaging protocols. Cerebrospinal fluid analysis revealed abnormalities in 24 of 41 cases (58.5%), with pleocytosis (> 5 WBC/μL) in 13 (37.1%) patients and elevated protein levels (> 45 mg/dL) in 23 (56.1%).

**TABLE 1:** Characteristics at diagnosis of patients with PACNS.

	All patients (*n* = 80)
Male	45 (56.3)
Age at diagnosis, median (range) years	39.35 ± 13.54
Clinical manifestations at presentation	
Headache	22 (27.5)
Sensory deficit	22 (27.5)
Dysphasia/aphasia	47 (58.8)
Seizures	31 (38.8)
Cognitive disorders	22 (27.5)
Others (visual impairment)	12 (15.0)
Diagnosed by biopsy	38 (47.5)
Lymphocytic	29 (36.3)
Granulomatous	8 (10.0)
Necrotising vasculitis	1 (1.3)
MRI enhancement	63 (78.8)
Enhancement duration > 6 months	27 (36.5)
Enhancement duration < 6 months	24 (32.4)
Meningeal involvement	16 (20)
No gadolinium enhancements	17 (21.6)
CSF abnormality	24/41^a^
WBC > 8 WBC/μL	13 (37)
Protein > 45 mg/dL	23 (56.1)
IL‐6 average	36.38 ± 117.11

*Note:* This table summarizes the demographic and clinical characteristics of the patient cohort at the time of diagnosis. Values are the number (%) or median (range).

Abbreviations: CSF, cerebrospinal fluid; MRI, magnetic resonance imaging; WBC, white blood cell.

^a^ CSF examination data were available for 41 patients. The remaining patients did not have CSF data examination at other hospital or had undergone previous brain tissue biopsies.

### 3.2 Treatment and Outcome

Longitudinal monitoring demonstrated a median follow‐up duration of 18 (3–96) months in our center, with 57 subjects (71.3%) maintaining clinical surveillance beyond 12 months. Treatment and outcome of patients was presented in Table 2. Of the total cohort, 75 patients (93.8%) received treatment: 21 (26.3%) were treated with glucocorticoids (GC) alone; 38 (47.5%) received GC in combination with intravenous cyclophosphamide (CYC); 6 (7.5%) received GC with rituximab; 5 (6.3%) received intravenous CYC alone; and 2 (2.5%) were treated with oral aspirin. GC pulse therapy consisted of intravenous methylprednisolone at 500–1000 mg daily for 3–5 consecutive days, with a median duration of oral corticosteroid therapy of 3 months (range, 3–11 months). Among the 80 patients, 27 (33.8%) discontinued all medications during follow‐up, whereas 46 (57.5%) received immunosuppressants as maintenance therapy. The most common regimens and their dosages were as follows: intravenous CYC at 1 g once monthly for 6 months; rituximab administered as two 500 mg infusions 14 days apart, followed by 500 mg every 6 months; mycophenolate mofetil (MMF) at approximately 1500 mg daily; azathioprine at approximately 100 mg daily; siponimod for maintenance at approximately 2 mg daily; and tocilizumab at 0.8 mg/kg intravenously once monthly.

**TABLE 2:** Treatment and outcome of patients with PACNS.

Initial treatment	
Steroid only	21 (26.3)
Steroid with cyclophosphamide	38 (47.5)
Steroid with Rituximab	6 (7.5)
Steroid with mycophenolate mofetil	3 (3.8)
Cyclophosphamide only	5 (6.25)
Aspirin	2 (2.5)
Patients receiving treatment	
Cyclophosphamide	48 (60)
Rituximab	16 (20)
Mycophenolate mofetil	5 (6.25)
Tocilizumab	1 (1.25)
Azathioprine	2 (2.5)
Siponimod	1 (1.25)
Cyclosporine	1 (1.25)
Adalimumab	1 (1.25)
Relapse	28 (35)
Median follow‐up month	18 (3–96)
Remission	62 (77.5)
mRS	
At diagnosis	2 (0–4)
At month 6	1 (0–4)
At month 12	1 (0–4)
At month 24	1 (0–5)
At last follow‐up	1 (0–5)
mRS ≤ 2	70 (87.5)

*Note:* This table summarizes the initial treatment regimens and subsequent therapeutic interventions, including patient functional outcomes at specified follow‐up intervals. Values are the number (%) or median (range).

Patients were categorized into four groups according to the therapeutic regimens. Group 1 consisted of 21 patients who received glucocorticoid monotherapy without induction or maintenance treatment with immunosuppressants. Group 2 included 6 patients who underwent induction therapy with glucocorticoids in combination with immunosuppressants but did not receive maintenance therapy. Group 3 comprised 41 patients who received both induction therapy with glucocorticoids and immunosuppressants, followed by maintenance treatment. Group 4 included 5 patients who did not receive glucocorticoid therapy but were treated with immunosuppressants for induction and maintenance. Seven patients could not be classified into any of the above groups (2 patients received aspirin monotherapy without glucocorticoids or immunosuppressants, and 5 patients received no treatment). Table 3 presents the treatment details and the relapse and remission status of patients in the four groups.

**TABLE 3:** Long‐term outcomes of PACNS patients based on induction treatment.

	Group 1 (*n* = 21)	Group 2 (*n* = 6)	Group 3 (*n* = 41)	Group 4 (*n* = 5)	*p*
GC					
Intravenous methylprednisolone pulses	20 (95)	6 (100)	38 (93)		0.631
Oral GC duration, month	3 (3–6)	3 (1–6)	6 (1.5–11)		< 0.01
Outcomes					
Follow‐up, month	24 (6–96)	21 (3–24)	16 (3–21)	11 (7–45)	0.335
Relapse	2 (10)	1 (17)	16 (39)	2 (40)	0.084
Relapse (n/y)	0.02 ± 0.07	0.15 ± 0.44	1.23 ± 0.62	0	0.53
Remission	21 (100)	5 (83)	32 (78)	4 (80)	0.040
mRS score					
At diagnosis	1 (0–4)	1 (1–4)	2 (1–4)	1 (1–2)	0.013
At month 6	1 (0–2)	1 (0–4)	2 (0–4)	1 (0–3)	0.036
At month 12	1 (0–2)	1 (0–4)	1 (0–4)	1.5 (0–3)	0.133
At month 24	1 (0–2)	4 (4)	1 (0–3)	4 (4)	0.074
At last follow‐up	1 (0–2)	1.5 (0–4)	1 (0–5)	1 (0–4)	0.075
mRS score ≤ 2 at last follow‐up	21 (100)	5 (83)	33 (80)	4 (80)	0.194

*Note:* This table presents the relapse, remission and functional outcomes across four patient groups under different initial and maintenance treatment regimens. Values are the number (%) or median (range).

Regarding treatment regimens, no significant differences were observed in the utilization of intravenous methylprednisolone pulse therapy among the four groups. However, a significant difference was noted in the duration of oral glucocorticoid maintenance therapy (*p* < 0.01). Group 3 had the longest duration of oral glucocorticoid treatment (median 6 months, range 1.5–11 months), which was significantly longer than that in Group 1 and 2.

Analysis of clinical outcomes demonstrated a trend toward differences in relapse rates across groups, with the highest relapse rate observed in Group 3 (39%, 16/41), followed by Group 4 (40%, 2/5); however, the overall difference did not reach statistical significance (*p* = 0.084). The long‐term remission rate was highest in Group 1 (100%, 21/21), which was significantly higher than that observed in the other three groups (*p* = 0.040).

Functional status was assessed using the modified Rankin Scale (mRS) score. At diagnosis, baseline mRS scores were significantly higher in Group 3 (median 2, range 1–4) compared with the other groups (*p* = 0.013). At the final follow‐up, the proportion of patients with an mRS score ≤ 2 showed no significant differences among the groups, indicating that most patients maintained favorable functional status at the last assessment.

### 3.3 Medication Changes

Among all enrolled patients, 13 experienced changes in their medication regimen during the follow‐up period, with 3 patients undergoing more than two modifications in immunosuppressive therapy. Among patients treated with cyclophosphamide, one patient switched to Siponimod after experiencing a relapse, four patients switched to Rituximab following relapse, and 5 patients continued with CYC maintenance therapy after relapse, following corticosteroid pulse treatment. One patient experienced persistent nausea as a side effect of CYC and switched to Rituximab for maintenance therapy. Another patient, after receiving six cycles of CYC with stable disease control, later transitioned to MMF for maintenance therapy.

Among patients treated with Rituximab, two were switched to CYC after relapse, and one was switched to CYC for B‐cell count failed to decrease to the desired level after Rituximab treatment. One patient treated with Tocilizumab switched to CYC after relapse. One patient treated with Azathioprine switched to CYC for hepatic function abnormalities. A patient on oral cyclosporine who relapsed was treated with a single cycle of cyclophosphamide and then resumed cyclosporine for maintenance therapy.

Given that therapeutic adjustments necessitate clinicians' professional judgment, standardized therapeutic monitoring is recommended prior to implementing treatment modifications, with timely regimen optimization based on disease progression. The core criteria for treatment modification established in this study encompass: (1) cumulative cyclophosphamide dose‐related toxicity; (2) drug‐related adverse events; and (3) failure to achieve predefined therapeutic goals. Notably, disease relapse does not constitute an absolute indication for treatment modification, given the delayed onset of therapeutic effects characteristic of immunotherapeutic agents and the common occurrence of transient disease fluctuations during clinical courses. Radiological changes serve as objective evaluation parameters, and when combined with an appropriate observation period (typically ≥ 6 months to allow adequate time for therapeutic response), prove valuable in differentiating genuine disease activity from natural fluctuations in disease course. Treatment modification is only initiated in cases where comprehensive clinical evaluation confirms persistent disease activity.

### 3.4 Functional Status

Longitudinal assessment of functional outcomes demonstrated progressive clinical improvement in the study cohort. MRS scores were prospectively recorded at predetermined intervals, with 77 (96.3%), 57 (71.3%), and 31 (38.8%) participants completing 6‐, 12‐, and 24‐month evaluations, respectively. Baseline evaluation revealed a median mRS score of 2 (0–4), with 56 subjects (72.7%) presenting favorable functional status (mRS ≤ 2). Serial assessments demonstrated significant functional recovery: median mRS decreased to 1 (0–5) at 6 months (64 patients [83.1%] with mRS ≤ 2), maintained at 1 (0–4) through 12 months (49 patients [86.0%]), persisting at 1 (0–5) at 24 months (28 patients [90.3%]). Final follow‐up data confirmed sustained improvement with median mRS of 1 (0–5) and 87.5% (70/80) achieving good functional status.

### 3.5 Factors Associated With Relapses

**FIGURE 2:**
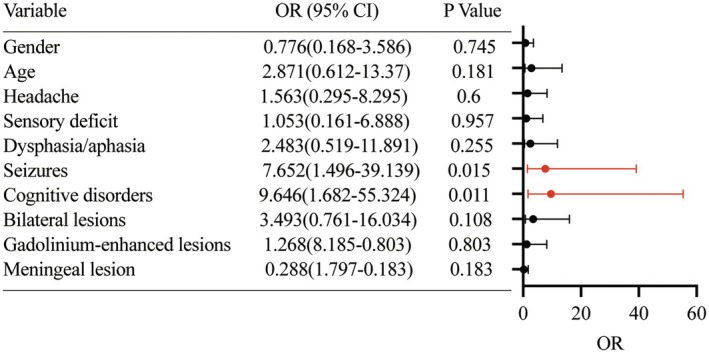
Displays the risk factors significantly associated with relapse in multivariable logistic regression analysis. Seizure manifestations and cognitive impairment were two predictors of relapse.

Longitudinal analysis of disease recurrence revealed 28 (35.0%) cases exhibiting ≥ 1 relapse event, including 8 subjects (10.0%) with multiple recurrences (> 2 episodes). Multivariable logistic regression analysis identified two independent predictors of relapse (Figure 2): seizure manifestations (OR [95% CI], 7.652 [11.496–39.139]; *p* = 0.015) and cognitive impairment (OR [95% CI], 9.464 [1.682–55.324]; *p* = 0.011). This indicates that patients with clinical manifestations of seizures or cognitive impairment may have a higher likelihood of disease relapses.

### 3.6 Factors Associated With Good Functional Status

**FIGURE 3:**
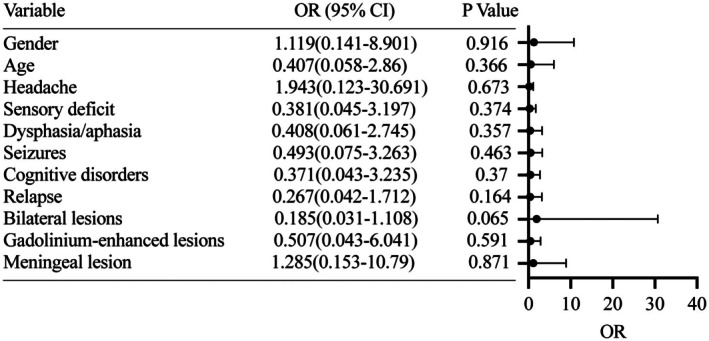
Displays the risk factors significantly associated with good functional status in multivariable logistic regression analysis. The presence of lesions was associated with good functional status at the final follow‐up.

Figure 3 analyzes the risk factors associated with good functional outcomes using multivariable logistic regression, showing that no significant risk factors clearly associated with poor prognosis were identified. Bilateral lesions (OR [95% CI], 0.185 [0.031–0.108]; *p* = 0.065) appeared more likely to be associated with poor prognosis, although this trend did not reach statistical significance.

## 4 Discussion

Our study demonstrates that, under standard treatment regimens—particularly those involving induction of remission followed by maintenance therapy with glucocorticoids combined with immunosuppressants—most patients achieve favorable functional outcomes.

PACNS has historically been associated with unfavorable clinical outcomes. Earlier epidemiological studies consistently reported that over 80% of affected individuals experience long‐term neurological deterioration and functional disability [3]. However, recent clinical investigations indicate that advances in diagnostic modalities and therapeutic strategies enable sustained disease remission in the majority of patients, consistent with the findings of our study [5, 9].

In our single‐center cohort of 80 PACNS patients (mean age 39.35 ± 13.54 years), histopathological confirmation was obtained in 38 cases (47.3%), a rate exceeding those reported in previous studies such as the Mayo Clinic (37.2%) and the French COVAC cohort (29%) [4, 9]. Subtype distribution analysis revealed granulomatous vasculitis in 8 cases (21.1%) versus lymphocytic vasculitis in 29 cases (76.3%), and necrotising vasculitis in 1 case (2.6%), diverging from established epidemiological patterns where granulomatous subtype predominates (58%) followed by lymphocytic variants (28%) and necrotising vasculitis (14%) [2]. It has been observed that the proportion of lymphocytic PACNS is higher in children PACNS [10]. Our cohort's younger mean age of onset compared to the Mayo Clinic cohort (39.35 vs. 48 years) may reflect a potential correlation between histopathological subtype and age at onset, with lymphocytic PACNS tending to occur at younger ages [4]. However, this study did not evaluate prognostic differences across pathological subtypes. Previous studies have been conducted to perform high‐throughput analysis of PACNS brain tissue biopsy samples. Whether the different histologic patterns represent more than one disease ultimately may permit development of novel diagnostic and therapeutic strategies [11]. Future studies should explore personalized treatment approaches based on pathological phenotypes to optimize PACNS outcomes.

Current treatment recommendations for PANCS primarily derive from observational studies or extrapolation from the management of systemic vasculitis. Glucocorticoid therapy should be initiated as soon as PACNS is diagnosed [12]. In our cohort, 85% of patients received methylprednisolone pulse therapy (500–1000 mg/day for 3–5 days) for induction remission treatment during the acute phase, a proportion higher than that reported in the French COVAC cohort (61%) [9], and lower than that in the Mayo Clinic cohort (96%) [13]. Notably, despite the absence of randomized controlled trials establishing glucocorticoid efficacy in acute PACNS, our cohort recorded no treatment‐related infections during high‐dose administration. For maintenance therapy, 52 patients (65%) received immunosuppressive regimens, with cyclophosphamide serving as the principal agent (*n* = 48, 60%). This utilization rate lies between those reported in the Mayo Clinic (45%) and AIIMS (48%) cohorts but is lower than in German (75%) and French COVAC (79%) cohorts [9, 13, 14, 15]. Given the cumulative dose‐dependent toxicities of CYC, no consensus currently exists on the optimal duration of therapy [16]. Additionally, 16 patients (20%) received rituximab, including 4 patients who switched to rituximab after relapsing following CYC therapy, with no subsequent relapses observed. These findings corroborate previous evidence supporting rituximab as a potential first‐line alternative to the more toxic CYC, particularly for patients who fail CYC therapy or have contraindications [17, 18].

During the treatment course, 13 patients (16.25%) underwent medication modifications, primarily due to disease relapse (*n* = 11) and adverse drug effects (*n* = 2). These changes were primarily based on clinician judgment‐based decisions reflecting current evidence gaps in PACNS management. Further research is needed to establish evidence‐based guidelines for optimizing therapeutic strategies in such scenarios.

Although PACNS is typically characterized as a disease with high relapse rates and significant mortality [19], 21 patients (26.25%) in our cohort were treated with glucocorticoids monotherapy during the acute phase and maintained on oral glucocorticoid without the additional immunosuppressants. Among these, only one patient experienced a single relapse. Importantly, six of these patients had histopathological confirmation of PACNS cases, suggesting that a subset of PACNS patients may exhibit a more benign disease course with a lower relapse risk, consistent with previous reports [20]. It suggests that glucocorticoids can be the appropriate treatment, with cyclophosphamide or a different immunosuppressant agent reserved for the treatment of relapses. This discrepancy may be attributed, in part, to the high heterogeneity of PACNS. Additionally, it is possible that the long‐term use of oral glucocorticoids, even in the absence of other immunosuppressive agents, contributed to this outcome. However, there is currently no randomized controlled trial evidence to confirm whether oral glucocorticoids are effective in preventing relapse. Current EULAR/ACR guidance [18] cautiously acknowledges glucocorticoid monotherapy for non‐progressive cases. Further studies are needed to elucidate the role of glucocorticoid monotherapy in the management of PACNS with a benign disease course.

Two patients in our cohort received oral aspirin, both achieving favorable disease control without experiencing relapse or harmful effects such as increased subsequent intracranial hemorrhage. The Mayo Clinic study intriguingly suggested an association between aspirin use and long‐term remission [13]. Several studies also indicate that aspirin may confer beneficial effects in the context of vasculitis [21, 22].

Among our cohort, 28 cases (35%) experienced at least one relapse during the disease. The relapse rate in contrast to the previous studies, where 27.7% in Mayo Clinic cohort [20] and 34% in French COVAC cohort [9], is lower than that reported in the Indian AIIMS cohort (59%) [14], reflecting variability likely due to cohort differences and shorter follow‐up duration at our center [23]. These findings underscore the necessity for long‐term clinical and imaging follow‐up for all PACNS patients, irrespective of relapse history. At final follow‐up, 70 patients (87.5%) in our cohort achieved a good functional status, which is higher than the rates reported in the French COVAC cohort (56%) [9] and the Indian AIIMS cohort (66%) [14], and also slightly higher than that of the Mayo Clinic cohort (85% for glucocorticoid monotherapy and 80% for combined CYC and glucocorticoid therapy) [13]. These findings highlight the variability in outcomes across different cohorts and underscore the importance of tailored treatment strategies and extended follow‐up to optimize patient outcomes.

We noted a trend toward higher recurrence rates in Group 3 and Group 4, while Group 1 achieved superior long‐term remission outcomes. Patients in Group 3 and Group 4 received maintenance therapy with immunosuppressive agents, despite differences in induction‐phase treatment strategies. The higher recurrence rates in these groups reflect a clinical tendency to preferentially prescribe immunosuppressive drugs for relapse prevention in patients with a prior history of recurrence, rather than indicating reduced treatment efficacy.

In our analysis, seizures (OR [95% CI], 7.652 [11.496–39.139]; *p* = 0.015) and cognitive dysfunction (OR [95% CI], 9.464 [1.682–55.324]; *p* = 0.011) demonstrated a statistically significant association with increased disease relapses. Conversely, we did not observe a significant association between the presence of MRI‐enhancing lesions or biopsy‐confirmed angiographically negative PACNS (small‐vessel subtype) and an increased frequency of relapse [13, 24]. These findings suggest that specific clinical manifestations, such as seizures and cognitive impairment, may serve as useful predictors of relapse in PACNS recurrence along with radiological findings. Future large‐scale, multicenter prospective studies incorporating neuroimaging and molecular biomarkers are warranted to develop robust prognostic models and elucidate underlying pathophysiological mechanisms of disease recurrence in this heterogeneous disorder.

Regarding functional outcomes, Bilateral lesions appeared more likely to be associated with poor prognosis, although this trend did not reach statistical significance. The Mayo Clinic cohort identified advanced age, stroke on MRI, and large‐vessel involvement as predictors of higher disability (mRS 4–6), whereas enhancing MRI lesions correlated with better outcomes [13]. The French COVAC cohort reported favorable functional outcomes primarily in patients who were receiving maintenance therapy [9]. Discrepancies across cohorts may stem from variations in treatment approaches and sample sizes. Based on our analysis of relapse risk and predictors of long‐term remission, patients presenting with seizures, cognitive impairment, or bilateral imaging lesions are considered to have a more severe disease course. For these high‐risk patients, corticosteroid induction therapy followed by maintenance immunosuppressive therapy is recommended. Regarding imaging surveillance, we suggest that low‐risk patients (without epilepsy or cognitive impairment) undergo imaging at least annually, whereas high‐risk patients (with concomitant epilepsy or cognitive impairment) undergo imaging at least every 6 months. Prospective studies with adequately powered designs are necessary to definitively ascertain risk factors for relapse and favorable functional status.

This study has several limitations inherent to its retrospective, single‐center design. The lack of a predefined protocol resulted in non‐standardized treatment strategies and follow‐up schedules, which may have introduced variability in patient outcomes. In addition, inconsistencies in medical records and imaging data associated with retrospective data collection may affect the reliability of the findings. It should also be noted that approximately half of the patients were diagnosed based on clinical criteria without histopathological confirmation; however, their long‐term follow‐up findings were consistent with a diagnosis of PACNS. Future multicenter, prospective studies are warranted to validate and extend these results.

In summary, our findings provide a comprehensive overview of relapse and prognosis in PACNS under current treatment regimens. Relapse remains common despite immunosuppressive therapy, underscoring the necessity of long‐term monitoring. Furthermore, the majority of patients can achieve remission and favorable outcomes, emphasizing the importance of aggressive management, especially in relapsing cases. Given PACNS heterogeneity and therapeutic complexity, further prospective research is essential to validate optimal immunosuppressive combinations, maintenance regimens, and treatment durations.

## Author Contributions

X.L.: methodology; formal analysis; writing – original draft. T.W.: writing – review and editing. L.Y.: data curation. F.‐D.S.: conceptualization; writing – review and editing; funding acquisition. H.W.: conceptualization; methodology.

## Funding

This research was supported by the National Natural Science Foundation of China (82320108007).

## Ethics Statement

This retrospective observational study was conducted using data from electronic medical records, with all personal identifiers removed to protect patient privacy.

## Conflicts of Interest

The authors declare no conflicts of interest.

## Data Availability

The de‐identified participant data used in this study are available from the corresponding author upon reasonable request for investigators following approval from the Institutional Review Board of Beijing Tiantan Hospital, Capital Medical University (Beijing, China).
